# A Mysterious Case of Primary Oral Tuberculosis in a 14-Year-Old Indian Female: A Diagnostic Enigma in a Case Report

**DOI:** 10.7759/cureus.85543

**Published:** 2025-06-07

**Authors:** Subhasish Burman, Asish K Das, Abhishek Khatua, Sandhya Tamilmani, Samrat Hembram

**Affiliations:** 1 Oral and Maxillofacial Surgery, Dr. R. Ahmed Dental College and Hospital, Kolkata, IND

**Keywords:** anti-tuberculosis therapy, evening fever, mycobacterium, paucibacillary, persistent pus discharge, tuberculosis, tuberculous osteomyelitis

## Abstract

Tuberculosis (TB), caused by *Mycobacterium tuberculosis*, is a chronic infectious disease that primarily affects the lungs but can involve other parts of the body, including the oral cavity, which leads to diagnostic challenges. This case report describes a 14-year-old female patient who presented with persistent swelling, pus discharge, and pain on the left side of the mandible for six months. Initial treatment, including incision and drainage, failed to resolve the symptoms. Imaging and histopathological examination revealed tuberculous osteomyelitis of the mandible. Despite negative microbiological results, the diagnosis was confirmed through the presence of granulomatous inflammation with giant cells on biopsy. The patient was subsequently referred for anti-TB treatment and showed significant improvement during follow-up. Oral TB, although rare, should be considered in the differential diagnosis of chronic oral lesions, especially when standard treatments fail. This case emphasizes the importance of comprehensive diagnostic approaches, including imaging and histopathology, which ultimately help initiate the correct treatment as early as possible.

## Introduction

In 1882, Robert Koch, a German scientist, discovered *Mycobacterium tuberculosis*, which causes tuberculosis (TB) [1]. TB is a chronic, systemic, granulomatous disease that can also occur in the lymph nodes, meninges, kidneys, bone, skin, and oral cavity. Other *Mycobacterium* species also cause TB, such as *Mycobacterium bovis* and atypical types [2]. TB infection is spread by direct inhalation of aerosolized bacilli contained in droplets of sputum; numerous droplets may be released while coughing, sneezing, or speaking. TB is classified into two types depending on the time of infection and type of response: primary TB and postprimary (i.e., secondary or adult) TB [3,4]. In the oral cavity, both types of TB can cause lesions [3]. Ordinarily, the occurrence of TB in the oral cavity is indefinite and can occasionally mimic cancer when the primary symptoms are absent [3]. Herein, we report a case of a non-healing ulcer, ultimately diagnosed as oral TB by oral biopsy after extensive testing.

## Case presentation

A 14-year-old female patient reported to the Department of Oral and Maxillofacial Surgery at Dr. R. Ahmed Dental College and Hospital, Kolkata, India, with chief complaints of swelling and pus discharge from the left side of the mandible for the past seven months (as of August 20, 2024). She had a history of mild pain in the lower left back tooth region for seven months and an initial rise in evening fever. She had no history of cough, weight loss, or loss of appetite. There was no history of TB among household members or close contacts.

**Figure 1:**
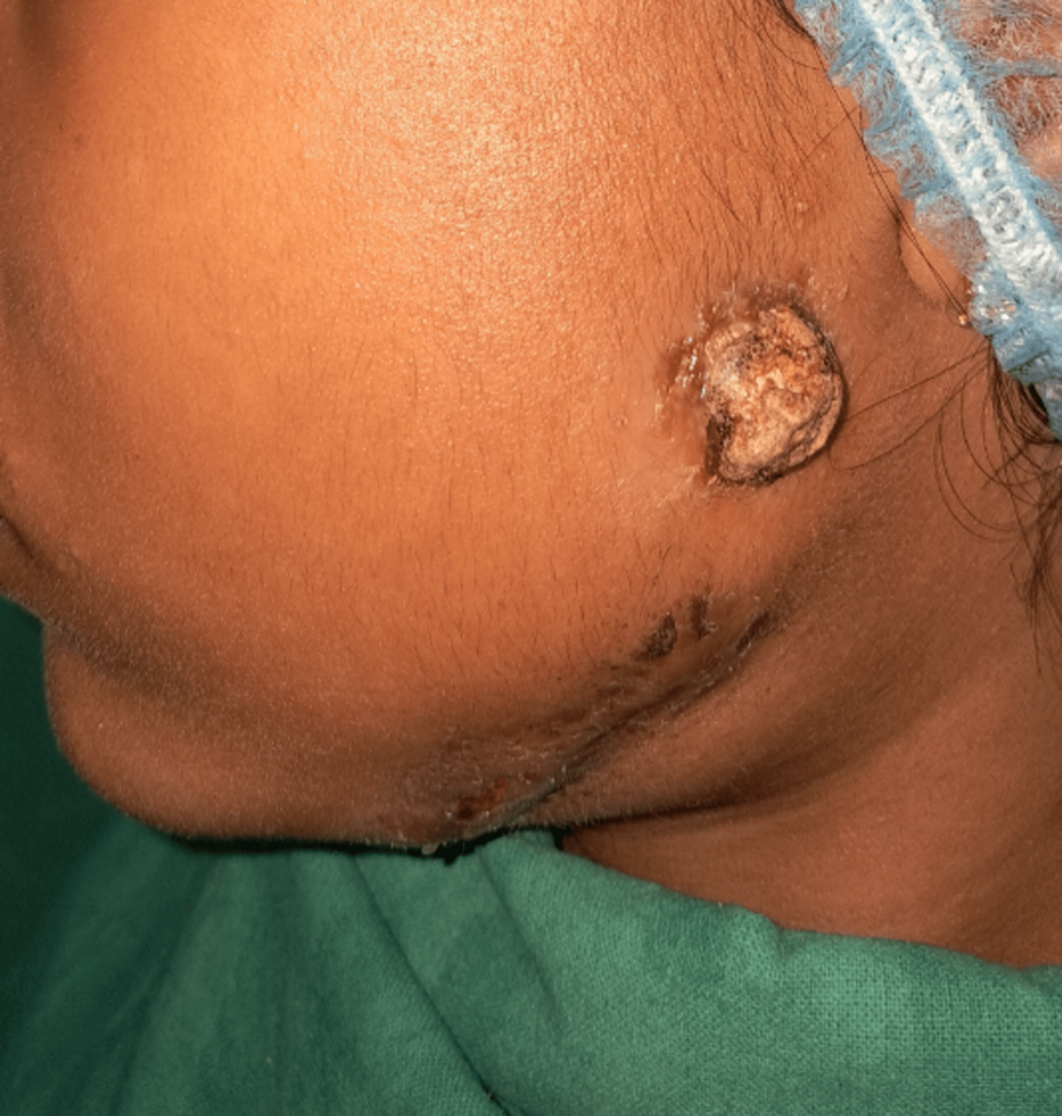
Pre-operative image

The patient underwent incision and drainage of the pus under antibiotic coverage in a private clinic on May 17, 2024, even though she is from a low socioeconomic background. However, she continued to experience swelling on the left side of the mandible and pus discharge in the left preauricular region, resulting in a reduced mouth opening of one finger width (Figure 1). A summary of symptoms is provided in Table 1.

**Table 1:** Summary of symptoms

S. No.	Symptom	Date of Event
1	Lower left side tooth pain	February 2024
2	Fever	February 2024
3	Swelling and pus discharge	May 2024
4	Incision and drainage	May 17, 2024
5	Persistent swelling and extraoral pus discharge	August 20, 2024

Contrast-enhanced computed tomography revealed that the ramus and coronoid process of the left mandible were affected by irregular osteolytic areas, extending from the 38 tooth follicle to the neck of the left condyle. All investigations performed are described in Table 2.

**Table 2:** Investigations performed Mtb*, *Mycobacterium tuberculosis*

S. No.	Investigation	Result
1	Fine needle aspiration cytology	Abscess
2	Cartridge-based nucleic acid amplification test	Negative for Mtb*
3	Ziehl-Neelsen stain	Negative for Mtb*
4	Pus culture	No growth
5	Posteroanterior view of chest X-ray	No abnormal results

**Figure 2:**
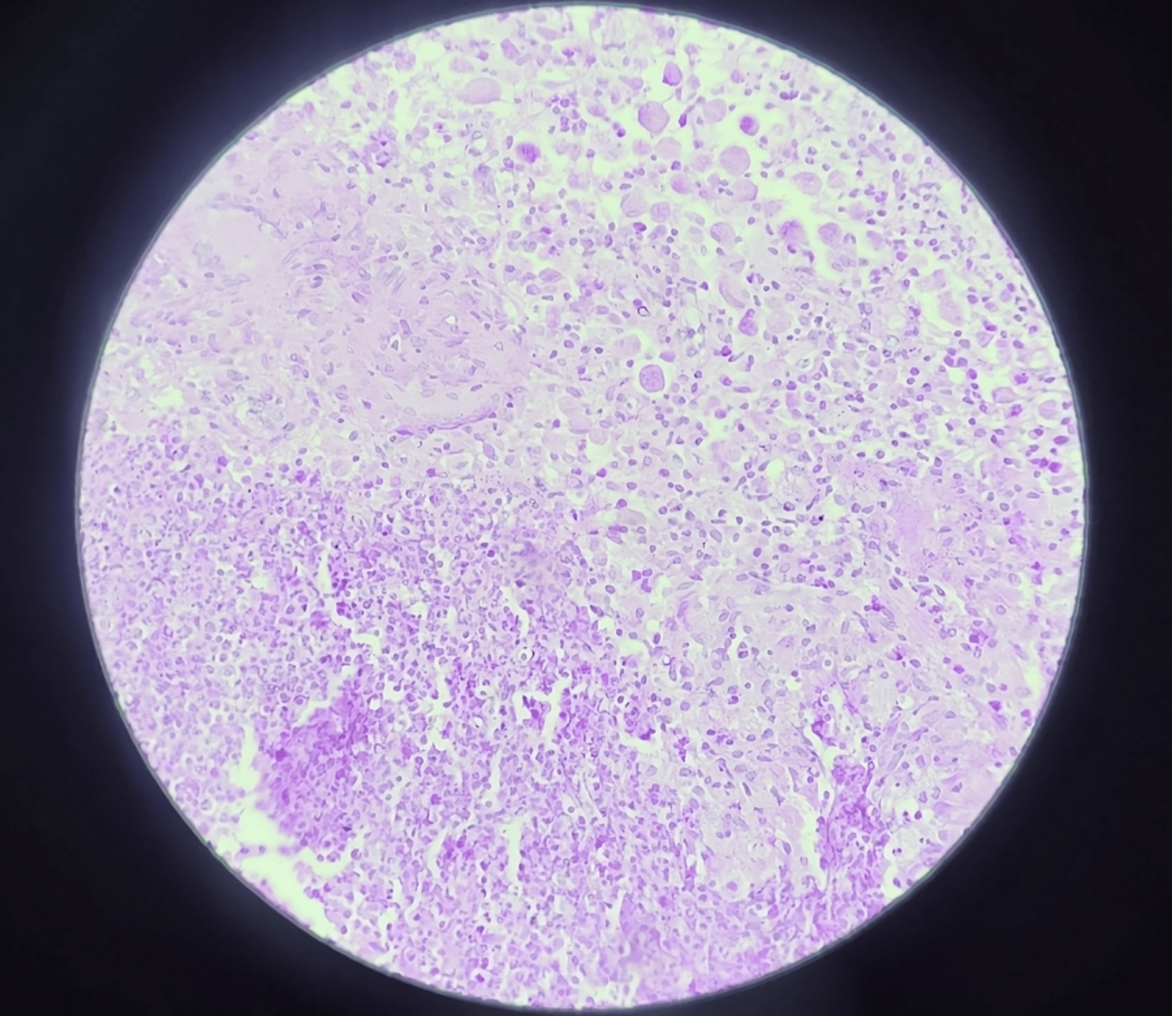
Histopathology slide

After achieving a two-finger mouth opening, and following routine blood investigations and serology testing, an intraoral biopsy was performed under local anesthesia. Histopathology revealed the presence of granulomatous inflammation and giant cells, leading to a diagnosis of tuberculous osteomyelitis (Figure 2), after excluding other granulomatous diseases such as sarcoidosis (which shows non-caseating granulomas), Crohn’s disease (which has microgranulomas), cat-scratch disease (which presents with star-shaped microabscesses), and tertiary syphilis (which shows granulomas with a central zone of necrosis).

**Figure 3:**
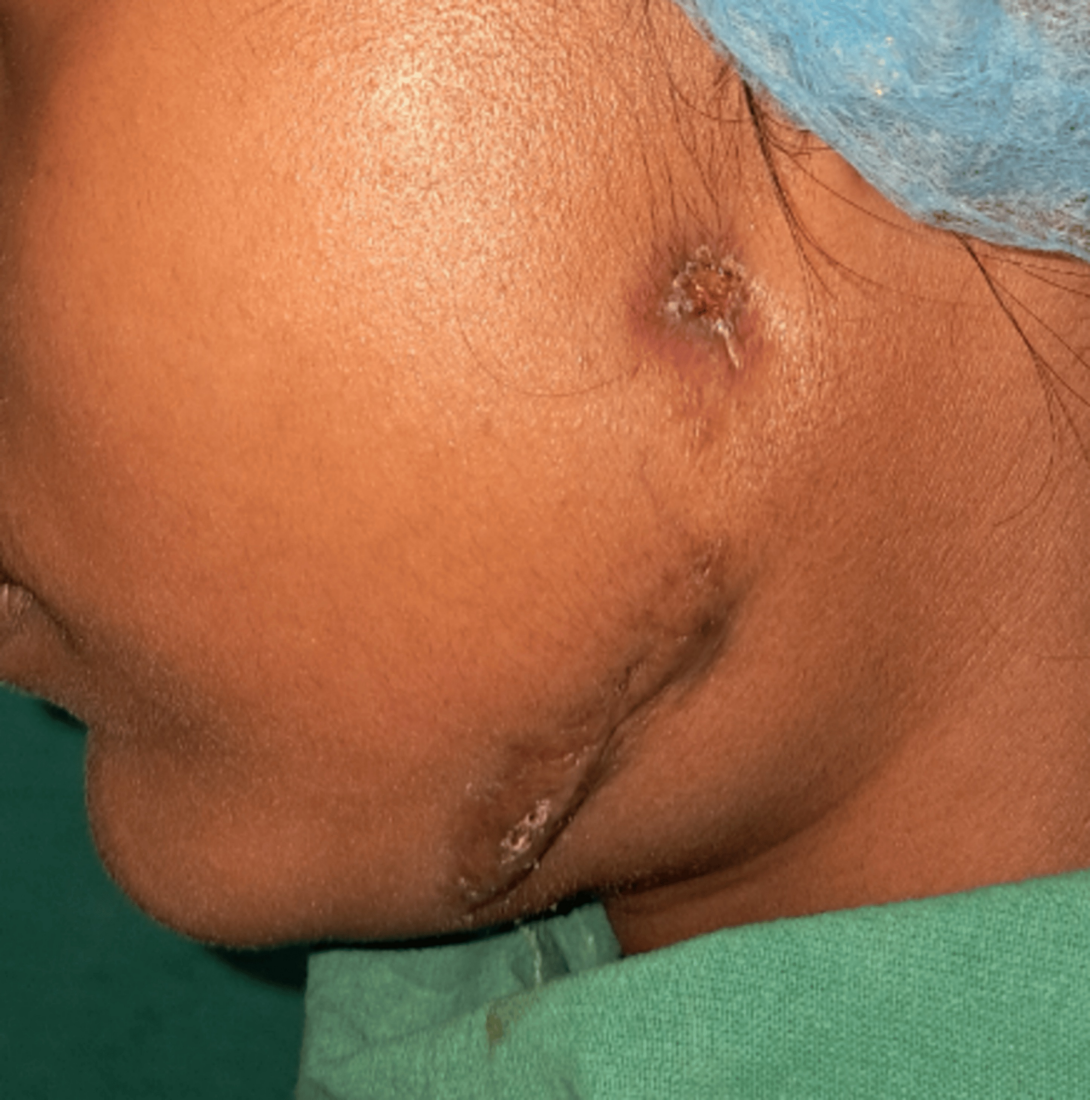
After one month of anti-tuberculosis treatment

**Figure 4:**
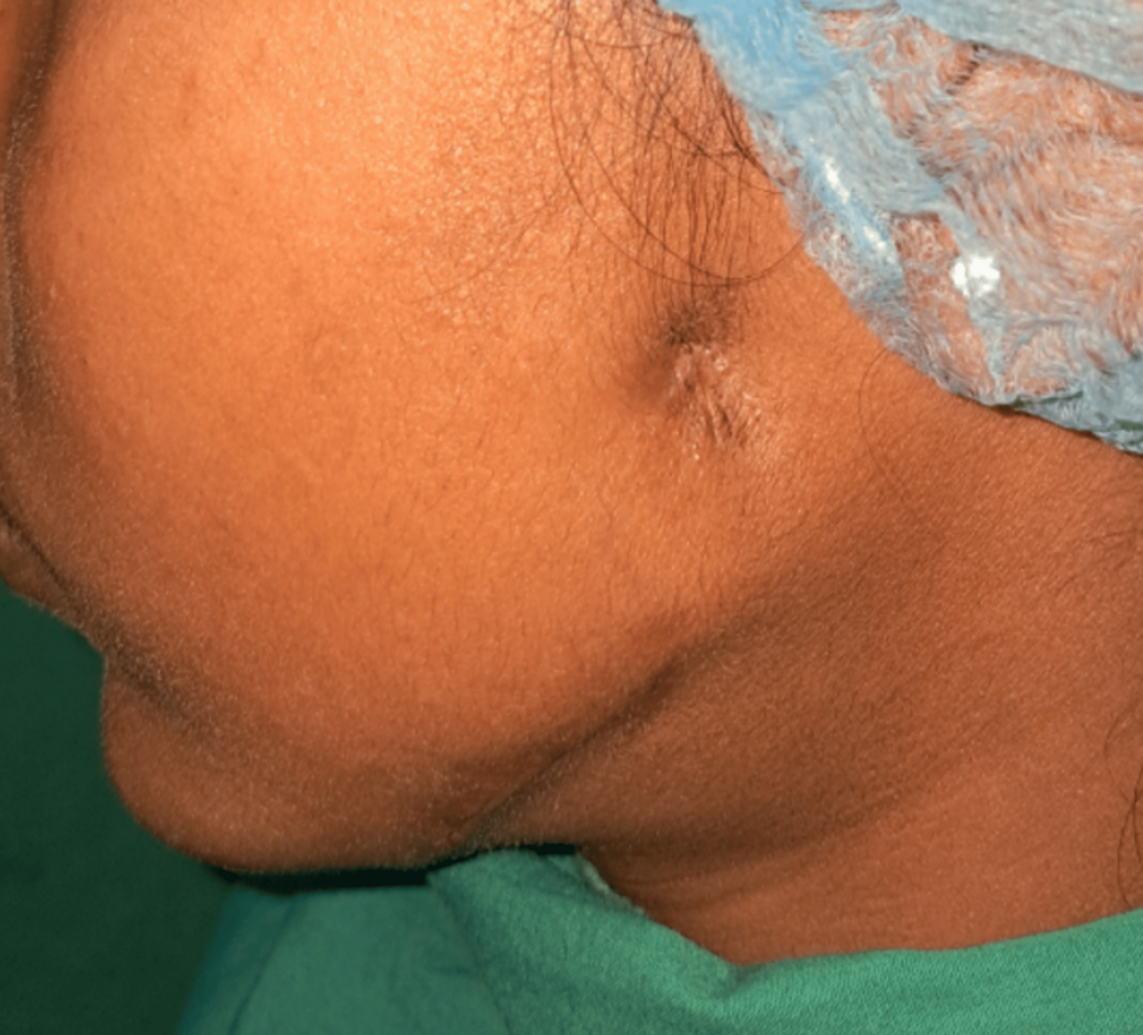
After three months of anti-tuberculosis treatment

The patient was then referred to the Department of Chest Medicine for further management and began anti-TB treatment. On periodic follow-ups during the first (Figure 3) and third months (Figure 4), good healing of the lesion was observed.

## Discussion

TB is a pandemic disease, with approximately 10.4 million people affected by the tubercle bacilli, according to a 2015 WHO report [1]. The prevalence of oral TB cases ranges from 0.5% to 1%. Primary TB consists of the Ghon focus (a subpleural focus of tuberculous pneumonia), along with enlarged hilar lymph nodes, which together form the primary complex. The incubation period is three to eight weeks from the time of contact with infected droplets [4]. Secondary TB usually exhibits necrosis and tissue destruction, ultimately leading to cavitation. Paucibacillary disease is seen in oral TB, and the bacterial count in saliva is markedly low, making confirmation through an acid-fast bacilli smear study difficult [1]. TB is mainly caused by self-inoculation with infected sputum; hematogenous spread also occurs [5-7]. The oral mucosa is somewhat resistant to the inoculation of tuberculous bacilli due to its intact epithelium [8]. However, when there is a breach in the mucosal barrier due to chronic irritation or inflammation - or other predisposing factors, including poor dental hygiene and dental extraction - it favors the colonization of organisms [9,10].

Oral TB can occur in the tongue and gingiva, and other sites such as tooth sockets, the floor of the mouth, the soft palate, the lips, and the buccal mucosa. Clinical presentation of TB lesions may be single or multiple in number, painful or painless infection, and generally ulcers appear as well-circumscribed with irregular borders [11-13]; they can begin as nodular-like structures, which may eventually develop like fissures, plaques, or vesicles, and then slowly increase in size, which leads to chronic infection [14]. Ulcers are normally surrounded by erythema, without induration, and usually satellite lesions can be found [11]. Differential diagnosis for oral TB may include traumatic ulcers, syphilitic ulcers, and malignancy, such as squamous cell carcinoma, lymphoma, and metastases. Histopathological investigation is the best method to diagnose oral tuberculous bacilli. Care must be taken to avoid misdiagnosis as other granulomatous lesions, such as sarcoidosis, Crohn’s disease, cat-scratch disease, and tertiary syphilis [1,15]. Andrade and Mhatre provided a classification system for orofacial TB [16].

Various manifestations of orofacial TB include tuberculous ulcer, tuberculous gingivitis, tuberculous dental periapical granuloma, tuberculous involvement of extraction sockets of teeth, TB of the maxillary sinus, tuberculous osteomyelitis of the maxilla and mandible, TB of the temporomandibular joint, tuberculous lymphadenitis, tubercular sialadenitis, and lupus vulgaris. Tuberculous osteomyelitis comprises less than 2% of overall TB, indicating its status as a rare entity [17]. Compared to the maxilla, mandibular involvement is more frequent [18]. The mode of infection is similar to that of other oral TB forms and may occur by direct or indirect transfer from infected sputum [19]. Chapote described four clinical forms of TB of the mandible [19]: the superficial or alveolar form, the deep or central form, the diffuse form, and the acute osteomyelitis form.

TB of the jaw leads to the formation of lumpy jaw, which appears as a painless, soft swelling. This chronic condition leads to single or multiple sinuses, which may be intraoral or extraoral. Sometimes, it results in a pathological fracture of the mandible due to bone necrosis or sequestration, which may also occur [18]. On radiographic examination, tuberculous osteomyelitis mostly presents as a diffuse type of bone loss and erosion of the cortical plate. It may also present as a mixed radiodensity appearance [16].

Treatment of orofacial TB is the same as standard antimycobacterial treatment regimens used for treating pulmonary TB. The treatment of oral TB lesions is identical to that of systemic TB. Currently, the most effective regimens require a combination of four drugs (isoniazid (INH), rifampicin (RIF), pyrazinamide (PZA), and ethambutol (ETO)) administered daily for two months, followed by an additional four months with three drugs (INH, RIF, and ETO). In 1997, WHO launched “Directly Observed Therapy, Short Course” in light of the difficulty of this regimen [1,20]. First-line antibiotics include INH 75 mg (H), RIF 150 mg (R), PZA 400 mg (Z), ETO 275 mg (E), and streptomycin 15-20 mg/kg. Second-line antibiotics include oral (Group I: PZA, ETO, and rifabutin) and injectable (Group II: kanamycin 15 mg/kg, amikacin 15 mg/kg, apreomycin 15 mg/kg, and streptomycin; Group III: fluoroquinolones like levofloxacin 10 mg/kg, moxifloxacin 10 mg/kg, and ofloxacin 15 mg/kg) options. Group IV oral bacteriostatic second-line agents include para-aminosalicylic acid 150 mg/kg, cycloserine 15 mg/kg, terizidone 250 mg, ethionamide 15 mg/kg, and prothionamide 15 mg/kg. Group V drugs, with an unclear role in the treatment of drug-resistant TB, include clofazimine 100 mg, linezolid 600 mg, thiacetazone 150 mg, amoxicillin/clavulanate 2-4 g, high-dose INH, imipenem/cilastatin 2 g, and clarithromycin 1000 mg/day.

New anti-TB drugs are being researched, and some have been approved, such as bedaquiline 400 mg for zero to two weeks, followed by 200 mg for 3-24 weeks, for the treatment of multidrug-resistant TB, which was approved in December 2012. Delamanid is the second drug that was approved by the European Medicines Agency in April 2014 for the treatment of drug-resistant TB [16,20]. Other new compounds are in the trial process.

## Conclusions

Oral TB, although rare, can present as a challenging clinical condition, often mimicking other pathologies such as malignancies or chronic infections. The case reported highlights the importance of considering TB in the differential diagnosis of oral lesions, especially when patients present with nonspecific symptoms like swelling and pus discharge, without clear signs of pulmonary involvement. The diagnostic approach, including fine needle aspiration, histopathology, and imaging studies, plays a crucial role in identifying tuberculous osteomyelitis of the mandible. In this case, the timely diagnosis and appropriate treatment with anti-TB drugs led to favorable outcomes, emphasizing the effectiveness of standard anti-tubercular therapy. This case underscores the significance of early detection, adequate investigation, and multidisciplinary management, as TB remains a major global health concern. With proper treatment, the prognosis for oral TB is good, and the risk of complications such as osteomyelitis or pathological fractures can be minimized. The case also highlights the need for heightened awareness among clinicians regarding the diverse presentations of TB, particularly in atypical sites like the oral cavity, which can sometimes be overlooked. This report is limited by its nature as a single case, which limits the accuracy of the assessment. Further studies, including larger case series, are needed to validate these observations.
